# *Praxe*: un dispositivo disciplinante portugués para la inserción al mundo académico

**DOI:** 10.18294/sc.2023.4537

**Published:** 2023-08-11

**Authors:** Lía Zóttola

**Affiliations:** 1 Doctora en Psicología. Profesora Asociada Regular, Universidad Nacional de Santiago del Estero, Santiago del Estero, Argentina. lia.zottola@gmail.com Universidad Nacional de Santiago del Estero Universidad Nacional de Santiago del Estero Santiago del Estero Argentina lia.zottola@gmail.com

Escribir sobre “la Academia”, sus contradicciones, sus modos de construir poder y las lógicas imperantes en algunos claustros, es un desafío reflexivo, sobre todo cuando se forma parte de un sistema que legitima espacios jerárquicos del saber y del poder, a veces inmerecidos o al menos exagerados. 

Se dice que las organizaciones son muestras de las diversidades, posibilidades y perversidades del funcionamiento de una sociedad. También la calle es un espacio organizador en el que se crean escenas cotidianas permanente y espontáneamente, donde ocurren fenómenos sociales que se invisibilizan, que se bañan de acriticidad, que potencian la naturalización de lo que no es natural, construyendo un sentido común que, en general, insensibiliza. 

Las trayectorias de las universidades europeas marcaron el rumbo y la génesis de las demás en el mundo occidental. En ellas se tejieron patrones culturales que han condicionado y conducido formas de producir conocimientos, de analizar, interpretar y organizar el mundo. Desde sus inicios estuvieron asociadas y sostenidas por castas acomodadas, sectores gozosos de privilegios eclesiales, de sangre o de bienes. 

En Portugal, hablar de “la Academia” y universidades pareciera no referirse a las mismas institucionalidades; sin embargo, están unidas: la una parece no existir sin la otra. La Academia es la organización estudiantil legitimada en el campo de la educación superior universitaria, que funciona en espacios universitarios y con la aprobación de las instituciones.

Entre las más antiguas, se encuentra la Universidad de Coimbra (fundada en 1290), que incluso se ubica geopolíticamente en un espacio de superioridad, arriba de la colina central de la ciudad. Na baixa (abajo), era el espacio destinado a los más pobres, los que más tarde serán identificados, desde la academia, como los “futricas”, la población que no accederá a los claustros universitarios.

Coimbra, la universidad más antigua de Portugal, cuenta en su historia con la Prisión Académica^1^, en la que los jóvenes estudiantes cumplían sus penas por desobediencias a las normas entre 1773 y 1832. Esta cárcel tenía, además, un fuero diferente al civil y funcionaba con un cuerpo policial propio, los “archeiros” bajo el mando de las autoridades universitarias, quienes tenían como misión el control del cumplimiento de las normas establecidas, tanto para los estudiantes como para los docentes. Incluso, podían impedir el acceso al campus al resto de la población que no perteneciera al mundo académico, a quienes identificaban como “futricas” (que se podría traducir como gente despreciable), siendo incluso hoy una de las categorías de clasificación de la población entre las jerarquías establecidas, en el Código da Praxe vigente de Coimbra^2^.

Esta cultura universitaria que se conserva por tradicionales rituales se expresa, por ejemplo, en el uso cotidiano de togas, galeras, capas, y uniformes académicos que les permiten a sus portadores identificarse para diferenciarse del resto de la población. Los docentes y los doctores las usan en determinadas celebraciones o rituales en los que la cultura se muestra atemporal: clases para abogados, imposición de cargos jerárquicos, defensas de tesis, imposición de reconocimientos y títulos honoríficos y académicos. Las y los estudiantes, pertenecientes a “la Academia”, usan trajes negros, con camisas blancas y capas negras. Originalmente era el uniforme para asistir a las clases, pero desde hace varias décadas los distingue junto al conjunto de prácticas y rituales de interacción conocidos como Praxe.

A poco de llegar a Portugal, escuché hablar de las “praxes” (prayes, pronunciado con acento castellano), me sonaba a praxis. Ignorante de este tipo de fenómeno, inexistente en mi país, imaginé que eran procesos formativos institucionalizados de prácticas transformadoras que permitirían proyectar los cambios políticos, sociales, humanos, que los espacios curriculares no lograban alcanzar. Algo de eso eran, pero no así. 

La Praxe es un conjunto complejo de prácticas que transforman la vida de cualquier sujeto, no cabe duda. Cada universidad tiene su Código de Praxe^2^^,^^3^. Las asociaciones académicas de estudiantes son organizaciones que se sostienen por tradiciones casi medievales, que se ordenan en jerarquías que devienen de la cronicidad estudiantil, la obediencia a las normas y la hegemonía del poder. En ellas, el Dux Veterano (al parecer suelen ser siempre hombres) es quien está en la cúspide de la pirámide por el mérito de haber permanecido más tiempo en su rol de estudiante o por tener mayor número de matriculaciones (para nosotros sería el estudiante más crónico). Algunos códigos de funcionamiento praxistas identifican a las y los estudiantes -de las categorías inferiores de la jerarquía de la asociación- con nombres de animales o sustantivos peyorativos “perro”, “bicho”, “puto”, etc. En otros códigos, se explicitan otros tipos de relaciones de subalternización que incluyen, por ejemplo, los servicios de tareas domésticas de los caloiros (los ingresantes) en las casas de los “académicos”^3^. 

La obediencia, la sumisión, el sadismo y la subordinación de los pares que, por supuesto, no los reconocen como tales, es la lógica que dinamiza “este ejército de capas negras”, de futuros profesionales universitarios que sostienen y justifican esta forma hegemónica de lo que pretenden considerar como procesos necesarios de “inclusión, socialización, integración e iniciación de los ingresantes novatos a la vida universitaria, a los que llaman caloiros”. 

La praxe ritualiza prácticas de sumisión, humillación colectiva de varones y mujeres, que se ejercen desde los estudiantes más veteranos hacia los más novatos. Tienen al menos tres periodos de sucesivos acontecimientos disciplinantes que van al compás del año académico: la apertura del año lectivo, las “latadas” (bautismos en los que se designa un padrino que detenta el control y al que se le debe obediencia), y la fiesta de la “queima das fitas”, celebrada desde 1850 (momento de las graduaciones y la ritualización a través del fuego donde queman las cintas -cada facultad tiene un color diferente- con las que simbolizan sus trayectos universitarios). Hoy es casi una atracción turística juvenil que es patrocinada por cervecerías, lo que hace que los ocho días que dura “este acontecimiento” (un día por cada facultad) la ciudad se transforme en un gran desparramo de jóvenes en estado de alcoholismo, a veces, grave.

Estos sucesos ocurren entre las aulas, los patios institucionales y en las calles de la ciudad. Son escenas que se montan y se desarrollan tanto en espacios públicos como privados. Basta explorar a través de Internet para encontrar cantidad de material visual y gráfico que da cuenta y permite registrar la dimensión social de estas manifestaciones naturalizadas de la vida universitaria portuguesa. Estas “novatadas” que se banalizan han costado, en ocasiones, la pérdida de vidas humanas^4^ o lesionadas para siempre. Sin embargo, tienen un grado de legitimidad que es difícil comprender a esta altura de la consolidación de derechos humanos, sociales, civiles, políticos del mundo occidental.

Las escenas se suceden en episodios en los que persiste el desfile de jóvenes encapuchados, cubiertos de capas negras, encorbatados, vestidos fuera de época y de estación climática. ¿Quiénes son?, ¿por qué visten así? Me llaman la atención. No son los protagonistas de las postales románticas de quienes tocan y cantan fados. También hay gente mayor con capas rojas, togas, sombreros, capas celestes y sombreros con adornos dorados: son los doctores. Los rituales de las universidades antiguas europeas no han aggiornado sus ropajes ni sus prácticas, tanto como sus ideas, teorías, y campos de conocimientos. 

Es difícil entender que estas prácticas se sostengan, y pese a que hubo periodos de prohibiciones, aún persisten, son legales y están legitimadas por la mayoría. Claro que también hay en la actualidad una minoría “antipraxe”^5^, casi siempre concentradas en “las Repúblicas”, que antes eran sitios de prácticas también praxistas. Hoy persisten y luchan como movimientos antipraxes, siendo marginalizados de los procesos y acontecimientos estudiantiles oficiales. Los rituales y festividades praxistas marcan de algún modo el cronograma académico en la ciudad. Basta leer algunos códigos para ver qué privilegios prometen a quienes formen parte de las praxes y de qué excluyen a los que deciden resistir y emanciparse.

¿Cómo romper esa estructura de obturación que anestesia la indignación frente a lo injusto, a lo cruel, a lo deshumanizante, a lo hegemónico? ¿Hay una sociedad fraccionada dentro y fuera de la universidad? ¿Hay una sociedad lega que acepta prácticas sancionables porque cree que es parte de las tradiciones de la élite intelectual a la que no pertenecen y, por tanto, no pueden cuestionar? Tantas contradicciones en una ciudad que parece vivir en varias épocas al mismo tiempo. 

Ante el asombro, una busca explicaciones, indagar, analizar, “solo quiero comprender”, decía Hanna Arendt. Pocos estudios se refieren a estos rituales de iniciación de estos espacios institucionales, dando cuenta de las percepciones que tienen los mismos jóvenes acerca de estas prácticas. Sin embargo, en los resultados de una investigación de hace un poco más de una década, los estudiantes valoran como positivos estos rituales, aunque refieren que debieran tener algunas modificaciones, pero no hacen mención a su cesación^6^. Eliseo Estanque, un docente de la Universidad de Coimbra, publicó un libro referido al análisis de las praxes^7^, en el que sostiene la hipótesis de que, en general, los jóvenes se muestran sumisos y sometidos a estas prácticas, del mismo modo en el que se muestran vaciados de conciencia política y cívica en lo cotidiano. Aceptar las prácticas praxistas es un modo de encontrar identidad y aceptación.

Hay contradicciones que surgen contundentes cuando se observa esta fragmentación de la realidad. Todo sucede en una especie de tinieblas a todas luces: la universidad de excelencia, la meca de los estudios sociales críticos, conserva estas prácticas seculares, mostrando la contracara de la producción académica por la que es referencia para el mundo sobre los estudios decoloniales-antipatriarcales-feministas-emancipadores-revolucionarios-, a veces hasta antisistema. Al mismo tiempo, se convive con la naturalización de los encapuchados, el enaltecimiento del “Dux Veterano” y el sadismo con los caloiros. Todo sucede simultáneamente en un tiempo esquizofrénico que ve sin ver, “ciegos que viendo no ven”^8^.

Las universidades son fábricas de grandes discursos y la calle es el escenario de las vivencias y la producción subjetiva de la vida, la realidad de la cotidianeidad de cualquier sociedad. La calle grita lo que la sociedad disimula.

Las praxes constituyen un fenómeno complejo y completo según Anibal Frias^9^ que, retomando a Marcel Mauss, lo referencia como un “objeto social total”, multidimensional con aristas culturales, políticas, lúdicas, simbólicas, gráficas, sonoras, etc.

Torbellinos de conceptualizaciones de las disciplinas sociales y humanas me surgen y me urgen como lluvia de ideas: sadismo, cuerpos y vidas dóciles, biopoder, hegemonía, dominación, alineación, obediencia ciega, subalternidad, patriarcalización, tutelaje, disciplinamiento, control… Se me ocurre que estas ideas podrían muy bien poner a dialogar a varios personajes de las ciencias. 

¿Frente a estas acciones sádicas, que diría Freud?^10^ Que el placer se centra en el sufrimiento físico, psíquico, en la humillación y el sometimiento de otro, cuando hay perversión. Los perversos no son los otros, somos nosotros mismos.

¿Qué agregaría Foucault a este comentario? Que estos cuerpos y vidas dóciles de los novatos necesitan ser disciplinados para lograr tener el control sobre ellos^11^, para sostener la soberanía de las acciones coercitivas que enaltecen al más veterano, no al más sabio. De esta forma y con la praxe como dispositivo de poder, se produce y reproduce un discurso de verdad que nadie va a osar cuestionar, ni siquiera el cuerpo docente. Así funciona la sociedad disciplinaria, se caracteriza porque el régimen de producción de verdad se construye a través de una red de dispositivos y aparatos que producen y regulan costumbres, hábitos y prácticas sociales. Es el mecanismo de biopoder más contundente que se puede ver fuera del ejército o las cárceles, simplemente en una pequeña ciudad universitaria.

Gramsci^12^^,^^13^ aportaría que este poder hegemónico cultural es una construcción de relaciones de orden político que genera consenso entre los grupos que se subordinan, que se subalternizan y se someten al orden establecido. Las praxes se sostienen porque hay consenso entre los estudiantes en que este es el camino a seguir, para lograr integración a la vida universitaria, para no ser marginalizados. 

Martín Baró^14^, en otra época y analizando las formas de violencia diría: los modos de violentar a otros necesitan estar autorizados. ¿Quiénes autorizan? Esos cuerpos docilizados, y también las instituciones que se preservan de tomar posición o partido, porque hay un orden establecido, instituido, que nadie tiene interés en romper. Esa es la tarea de la psicología social, diría Moscovici.

Si pensamos en Hannah Arendt nuevamente, diríamos que procesos violentos considerados “autorizados” producen efectos colectivos y, desde la psicología sociocomunitaria se pueden entender como formas cotidianas que, de modo imperceptible, van dejando huellas de opresión social en los modos de funcionamiento de la vida^15^. La “banalidad del mal”^16^ conceptualiza una forma de perversión de la conducta de una persona que no encaja con los modelos con que habitualmente se representa la maldad humana. A propósito de Adolf Eichmann: “un hombre corriente que hace cosas atroces”^16^, se refiere a la posibilidad de que una persona, con características “normales”, es capaz de provocar daño a otras. ¿Quién diría que en las universidades hay tanta maldad?

La hipótesis que se plantea entonces es que el discurso social académico justifica “la banalización del sufrimiento social”, legitima la desigualdad e inequidad provocando efectos alienantes, despersonalizantes y desafiliadores. Así, los sujetos pierden su capacidad de agencia porque se objetivan en la lógica del que manda, del que decide cuando visibilizarlos e invisibilizarlos a través de formas de silenciamiento del malestar y la desigualdad social, procesos que metamorfosean -en el sentido kafkiano- la condición humana^16^. 

¿Cuántas formas de sometimientos admite la academia? ¿Cómo se construyen y como se deconstruyen? ¿Quiénes establecen los tiempos o los plazos para sostener una figura poderosa del régimen disciplinario? ¿Qué le hace caer? ¿Habrá oportunismo? ¿Habrá castigo? ¿Es auténtica búsqueda de transformación social? ¿Qué se busca en realidad? Nada se sostiene ni se genera sin la presunción de beneficio. ¿Cuál es el beneficio? ¿A qué estamos dispuestos para obtener privilegios? ¿Dónde caben las responsabilidades individuales y las colectivas?

¿Tal vez se pueda presagiar el peligro de romper el acuerdo tácito de los silencios? ¿Los que callaban, hablarán? En esos casos, hasta las paredes podrán perder la sordera y la mudez. Será necesario que emerjan de las grietas nuevos discursos de verdad para que se propaguen en esta época en pocos segundos como un virus que viaje a velocidades de la luz y del sonido aprovechando la tecnología para repensarse.

¿Será posible debatir abiertamente sobre los regímenes de verdad, la cultura de la violencia, al menos simbólica, dentro de las instituciones educativas, de las “altas casas de estudios superiores”? ¿Será posible pensar en una democracia de la vida cotidiana? Democracia como forma de vida, ¿no solo como régimen de administración de un Estado? Siguiendo a Elísio Estanque^7^, el camino de la abolición de las praxes puede venir de la mano de una cultura democratizante de sensibilización de conciencia ciudadana entre los jóvenes. ¿Será posible?
